# Precision Delivery of Active Compounds from Edible and Medicinal Plants via Gut Microbiota Targeting: A New Paradigm for Cancer Immunotherapy

**DOI:** 10.3390/nu17223591

**Published:** 2025-11-17

**Authors:** Lu Peng, Long Wang, Yingtong Zhao, Peng Yu, Hongmei Yang

**Affiliations:** 1 Public Experimental Center, Changchun University of Chinese Medicine, Changchun 130117, China; 2 School of Biological Sciences, University of California San Diego, La Jolla, CA 92093, USA; 3 College of Pharmacy, Changchun University of Chinese Medicine, Changchun 130117, China

**Keywords:** cancer immunotherapy, gut microbiota, bioactive components, edible and medicinal plants, drug carriers

## Abstract

The immune system plays a pivotal role in the progression of tumors. Recent advancements in immunotherapies, notably CAR-T cell therapy and checkpoint inhibitors, have markedly improved clinical outcomes. However, a significant proportion of patients continue to experience treatment resistance, posing a persistent and formidable challenge. The gut microbiota has been established as a critical determinant of responses to immunotherapy. Enriched with bioactive components such as polysaccharides, (poly)phenols, and flavonoids, edible and medicinal plants (EMPs) exhibit significant potential to enhance host immunity by reshaping the gut microbiota, increasing the production of microbiota-derived metabolites (e.g., short-chain fatty acids), strengthening the intestinal barrier, and reducing intestinal inflammation. The bioactive components derived from EMPs not only demonstrate substantial pharmacological activities but also serve dual roles: functioning either as inherent drug carriers or as effective modifiers for existing carrier systems, which facilitates targeted drug delivery to specific sites such as the liver and intestinal, enhancing therapeutic efficacy. In summary, this review highlights that bioactive components from EMPs hold significant promise for enhancing cancer immunotherapy by modulating complex interactions with the gut microbiota.

## 1. Introduction

Cancer poses a significant threat to human health, with GLOBOCAN 2022 data revealing approximately 20 million new cases and 9.7 million deaths globally with its mortality rate ranking second globally [1]. Traditional cancer treatments, such as surgery, radiotherapy, chemotherapy, and targeted therapy, often lead to tumor recurrence and unfavorable prognosis. Immunotherapy targets immune cells and their interactions with tumor cells [2] and can treat metastatic malignant tumors and prolong the survival time of patients with good prognosis. However, due to the immunosuppressive tumor microenvironment (TME) and the immune escape mechanism of tumor cells, immunotherapy has limited efficacy in tumors [3]. At the same time, in the process of cancer immunotherapy, the emergence of drug resistance, immune-related adverse reactions and heterogeneity make immunotherapy face great clinical challenges.

The gastrointestinal tract contains 60–80% of the immune cells, which are crucial for maintaining intestinal homeostasis [4]. Research demonstrates that gut microbiota and metabolites not only modulate intestinal immunity but also exert influence on systemic cancer immunomodulation [4,5]. Recent studies show that the efficacy of cancer immunotherapy, encompassing immune checkpoint blockade (ICB), CAR-T cells, and cancer vaccines, varies significantly among individuals, which is related to the gut microbiome [6,7,8,9,10]. Therefore, gut microbiota represents a highly promising target for precision cancer immunotherapy.

Edible and medicinal plants (EMPs), as an indispensable and important part of traditional Chinese medicine, have functions of both nutritional and pharmacological. Due to high safety, minimal side effect, abundant functional components including polysaccharides, flavonoids, and saponins and multi-pathway pharmacological, EMPs display unique advantages in the field of cancer treatment, improving quality of life for patients [11]. Emerging evidence demonstrates that EMPs can modulate antitumor immune responses through gut microbiota regulation [12]. This interaction underscores the intricate interplay between active compounds de-rived from EMPs, the gut microbiome, and the cancer immune system. By delving into this crosstalk, researchers gain invaluable insights into the development of innovative immunotherapeutic strategies. Furthermore, the article examines current research advancements in utilizing active compounds from EMPs to engineer drug delivery systems for tumor therapy, offering translational insights for clinical applications.

## 2. Bioactive Compounds from EMPs and Regulation of Gut Microbiome

**Figure 1:**
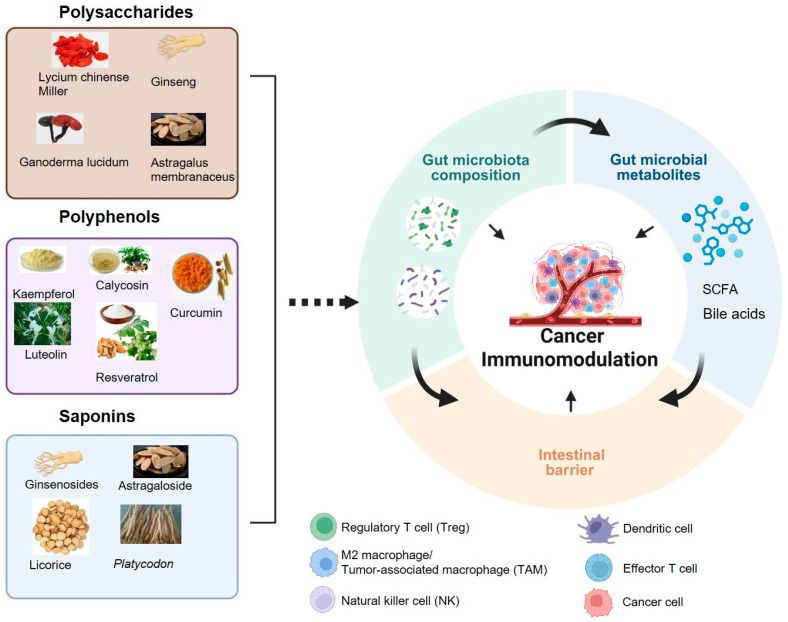
The Interaction between bioactive compounds from edible and medicinal plants and gut microbiota in cancer immunomodulation. Created with biorender.com.

EMPs have been widely utilized in China, Korea, Japan, and other countries for treating various diseases, including cancer [13], inflammatory disorders [14], and neurodegenerative disease [15,16]. According to chemical structures, bioactive compounds from EMPs are categorized as polysaccharides, flavonoids, saponins, and (poly)phenols, alkaloids, terpenoid, phenolic compounds, coumarin, essential oil and volatile oil. Extensive research has shown that polysaccharides, saponins, and (poly)phenols found in MFH plants enhance anti-tumor immunity by directly or indirectly modulating immune cell activity and the gut microbiota [12,17,18,19] (Figure 1).

### 2.1. Polysaccharides

Polysaccharides are a class of natural bioactive compounds abundantly present in EMPs, including *Astragalus membranaceus* [20], *Panax ginseng* [21,22], and *Lycium chinense* [23,24], demonstrating unique anti-tumor activities. Increasing evidence demonstrates that polysaccharides play a crucial role in maintaining gut microbial homeostasis by regulating both the structural composition and metabolic activities of the gut flora, preserving intestinal barrier integrity, and ultimately enhancing gut immunity [25,26]. Ginseng polysaccharides (GPs), bioactive components of *Panax ginseng*, enhanced PD-1 blockade therapy by modulating gut microbial metabolism and composition, which collectively increased CD8^+^ T cell recruitment while inhibiting Treg-mediated immunosuppression. Furthermore, GPs promote the abundance of *Parabacteroides distasonis* and *Bacteroides vulgatus*, bacterial genera crucial for ginsenoside biotransformation, which was significantly associated with therapeutic response and prognosis. Clinical investigations have demonstrated that GPs improves therapeutic response to PD-1 checkpoint blockade, potentially overcoming immunotherapy resistance [27]. The water-soluble polysaccharides extracted from the spore bodies of Ganoderma lucidum polysaccharides (GLP) inhibit the TLR4/MyD88/NF-κB signaling pathway, improving the microbial flora imbalance induced by AOM/DSS, increasing the production of short-chain fatty acids, and alleviating endotoxemia. In addition, GLP can significantly improve the intestinal barrier function, inhibit the infiltration of macrophages, and suppress colonic inflammation and tumor occurrence [22]. In addition, emerging research indicates that polysaccharides such as *Lycium barbarum* polysaccharides [28], *Lentinula edodes* polysaccharides [29,30] and *Astragalus* polysaccharides (APS) [31] modulate gut microbiota and their derived metabolites, promoting the restoration of immune function. These findings demonstrate that polysaccharides can serve as adjuvants to improve intestinal and immunosuppression.

### 2.2. (Poly)Phenols

(Poly)phenols, renowned for their robust antioxidant and anti-inflammatory benefits, are one of the most important and widely occurring bioactive components in EMPs. Flavonoids, phenolic acids, lignans, and stilbenes are the four main types of (poly)phenols [32]. Among these, flavonoids are the most abundant, followed by phenolic acids.

Flavonoids exhibit diverse biological activities and pharmacological effects. Their diverse structures, including isoflavones, neoflavonoids, flavones, flavonols, flavanones, flavanonols, flavanols, and chalcones, endow them with potent antioxidant, anti-inflammatory, and anti-tumor properties [33]. Accumulating research suggests that flavonoids extracted from EMPs, such as kaempferol (a flavonol) and calycosin (an isoflavone from *Astragalus membranaceus*), can enhance cancer immunotherapy by activating antitumor immune responses through remodeling the gut microbial diversity and modulating microbial metabolites [34,35,36]. Inflammatory bowel disease (IBD) is a persistent and debilitating condition characterized by chronic intestinal inflammation with the potential to progress to colorectal cancer (CRC) [37]. Luteolin, a bioactive flavonoid derived from EMPs such as *honeysuckle* (*Lonicera japonica*), *astragalus* (*Astragalus membranaceus*), and *angelica* (*Angelica sinensis*), demonstrates significant therapeutic effects against IBD [38,39]. Its mechanisms of action include: (1) modulating T-cell subset differentiation [40], (2) downregulating pro-inflammatory mediators, (3) activating antioxidant pathways [41,42], (4) enhancing gut microbiota composition [43,44], and (5) restoring intestinal mucosal barrier function through specific molecular signaling pathways [45]. These multifaceted actions collectively ameliorate IBD symptoms and consequently suppress CRC development. However, the majority of (poly)phenols that escape gastrointestinal absorption reach the colon directly, and are metabolized by gut microbes into a spectrum of smaller phenolic acids. These metabolites exhibit greater biological activity and higher absorption efficiency. For example, ellagic acid is converted into urolithins, which significantly enhance gut barrier function, reduce inflammation, and create a systemically favorable environment for antitumor immunity [46]. Furthermore, resveratrol, a natural stilbenes compound enhances antitumor immune responses by modulating the abundance and composition of intestinal microbiota, ultimately enhancing the outcome of cancer immunotherapy [47].

Additionally, other polyphenolic compounds such as curcumin can remodel the gut microbiota composition by enriching beneficial bacteria, which are closely associated with the maintenance of intestinal barrier integrity, anti-inflammatory effects, the production of beneficial metabolites and tumor microenvironment remodeling [48,49,50].

### 2.3. Saponins

Saponins are a constitute a diverse class of bioactive compounds ubiquitously present in EMPs. These structurally complex phytochemicals exhibit diverse biological activities attributable to their intricate chemical architectures, including immunomodulation [51,52], cardiovascular protection [53,54], antioxidant effects [55,56], and antitumor properties [57,58,59]. Based on their aglycone structures, saponin family, two primary categories can be distinguished: triterpenoid saponins (such as *ginseng*, *licorice*, *astragalus*, and *platycodon*) and steroidal saponins (like *ophiopogon japonicus* and *Chinese yam*). Among these, triterpenoid saponins are particularly notable for their immunomodulatory and antitumor activities, such as ginsenoside and astragaloside. Emerging evidence suggests that multiple ginsenosides (such as Rh2 [60], Rg1 [61] and Rk3 [62]) regulate gut microbial diversity and promote anti-tumor immune responses, inhibiting tumor growth. Rk3, in particular, has demonstrated remarkable potential in ameliorating gut microbi-ota dysbiosis by promoting the enrichment of beneficial bacteria like *Akkermansia muciniphila* and *Barnesiella intestinihominis*. And eliminating pathogenic *Desulfovibrio* species, Rk3 restores intestinal barrier integrity. Mechanistically, Rk3 modulates chemokine and inflammatory cytokine production through regulation of innate lymphoid cells and T helper 17 (Th17) cell signaling pathways, significantly suppressing colonic tumorigenesis [62]. Moreover, recent studies revealed that Astragaloside IV(AS-IV) exhibits potent immunomodulatory and gastrointestinal protective effects by alleviating mucosal inflammation and restoring intestinal barrier integrity during different stages of IBD progression to colorectal cancer. AS-IV exerts their therapeutic effects through multiple mechanisms, including targeting key immune signaling pathways (like NF-κB and PPAR) and promoting M1 macrophage polarization, suppressing tumor growth and metastasis via multi-targeted actions, which offers novel strategies for future drug development [63].

## 3. Synergistic Effects of the Bioactive Compounds from EMPs on Immunotherapy via Gut Microbiota Modulation

**Figure 2:**
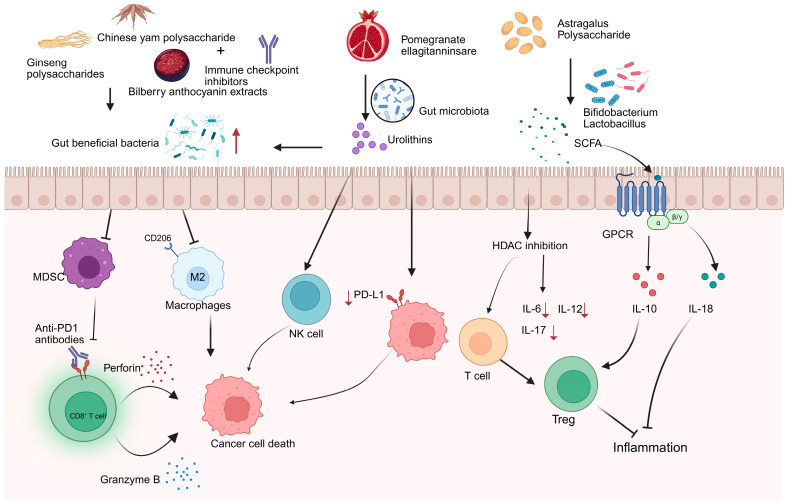
The interaction bioactive compounds from edible and medicinal plants and gut microbiota in cancer immune response. Created with biorender.com.

**Figure 3:**
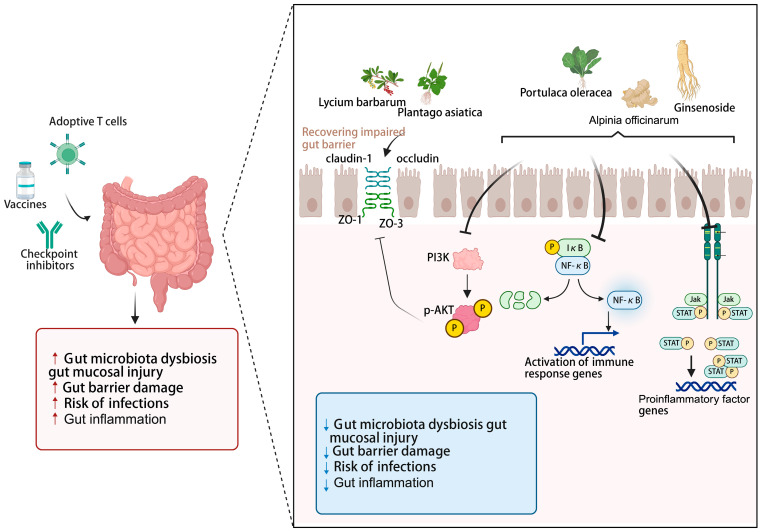
Natural bioactive compounds from edible and medicinal plants improve intestinal barrier dysfunction and alleviate intestinal inflammation in cancer immunotherapy. Created with biorender.com.

To counteract tumor immune evasion during cancer progression, various immunotherapies have been developed [2]. However, tumor immunotherapy faces substantial limitations and challenges. Immune checkpoint blockade therapies, notably PD-1 and PD-L1 blockers, demonstrate clinical efficacy in only approximately <30% of patients, with a subset of responders eventually experiencing disease recurrence [64]. Meanwhile, chimeric antigen receptor (CAR) T-cell therapy exhibits limited therapeutic potential against solid tumors [65]. Furthermore, immunotherapeutic interventions are often accompanied by severe immune-related adverse events and can lead to the development of treatment resistance. Recent preclinical and clinical studies have brought the gut microbiota into the spotlight, revealing its pivotal role in modulating the patient’s response to immunotherapy [8,66,67]. Research indicates that the gut microbiome-derived metabolites and secreted molecules enhance immunotherapy efficacy [68,69]. With advantages including natural derivation, multi-target modulation, and low toxicity, bioactive compounds from EMPs can synergize with immunotherapy by modulating gut microbiota, which reduces treatment toxicity while improving antitumor efficacy, leading to better patient outcomes. Emerging research reveals that active ingredients from medicinal foods potentiate cancer immunotherapy via modulating gut microbiota composition, promoting the production of beneficial metabolites such as short-chain fatty acids (SCFAs) (Figure 2), and improving intestinal barrier function to reduce inflammation (Figure 3).

### 3.1. Restructuring of the Gut Microbiome

Emerging evidence suggests that specific gut bacteria, notably *Bacteroides fragilis*, *Bacteroides thetaiotaomicron*, *Bifidobacterium* species, *Akkermansia muciniphila*, and *Faecalibacterium* spp., may improve the outcome of cancer immunotherapy, as observed in preclinical studies and patient cohorts [8,9,70,71]. Consequently, therapeutic strategies targeting gut microbiota modulation have been proposed to enhance treatment efficacy in oncology. Among immunotherapeutic approaches, immune checkpoint inhibitors (ICIs) constitute the most extensively studied class of immunotherapeutic agents [72,73,74]. However, their low response rates in cancer patients remain a major therapeutic challenge.

As the dominant effector population in anti-tumor immunity, CD8^+^ T cells have shown remarkable therapeutic efficacy in various immunotherapeutic strategies. Anti-PD-1 antibodies blockade activated CD8^+^ T cells to secrete perforin and granzyme B, mediating tumor cell death [75,76]. Modulation of the microbiota-immune axis by bioactive compounds from EMPs represents a key mechanism for improving ICI efficacy. GPs, for instance, enhance immune surveillance. They achieve this by elevating *Lachnospiraceae* abundance, an effect that suppresses myeloid-derived suppressor cells (MDSCs) and subsequently facilitates the infiltration and activation of CD8^+^ T cells [77,78]. When combined with αPD-1 monoclonal antibody (mAb) therapy, GPs demonstrate an additional layer of benefit. They reshape the gut microbiota profile, leading to improved sensitivity to anti-PD-1 immunotherapy in patients with non-small cell lung cancer (NSCLC) [27]. In addition, recent research showed that Chinese yam polysaccharide (CYPs), derived from medicinal food plants, exhibit immunomodulatory and antitumor properties. 16S rRNA sequencing revealed that combined treatment with CYPs and αPD-1 monoclonal antibody (mAb) enriched beneficial bacteria (e.g., *Clostridium_UCG-014* and *Actino-bacteria*), while simultaneously reducing the abundance of pathogenic bacteria including *Enterobacteriaceae* and *Desulfovibrionaceae*. This shift in gut microbiota composition was accompanied by modifications within the tumor microenvironment (TME). Specifically, the combination therapy suppressed immunosuppressive M2 macrophages (CD206+ subset) and enhanced the infiltration of cytotoxic CD8^+^ T cells. Consequently, patients with colorectal cancer (CRC) exhibited an improved response to anti-PD-1 immunotherapy [79]. Bilberry anthocyanin extracts enhanced the efficacy of ICIs by elevating the relative abundance of *Clostridia* and *Lactobacillus johnsonii* and increasing overall microbial community diversity [80].

Based on the aforementioned findings, we conclude that bioactive components de-rived from EMPs enhance the efficacy of tumor immunotherapy through dual mechanisms: on one hand, by modulating the composition of the gut microbiota (promoting the proliferation of beneficial bacteria and inhibiting the growth of harmful ones), and on the other hand, by regulating the immunosuppressive tumor microenvironment. This discovery opens new avenues for microbiota-targeted cancer therapy, fully demonstrating the application potential of EMPs bioactive in the field of immune regulation.

### 3.2. Microbial Metabolites

Metabolites derived from gut microbiota reshape the tumor microenvironment [81,82,83]. These metabolites boost the efficacy of immunotherapy by activating the immune response, effectively eliminating tumor cells, and overcoming drug resistance. Furthermore, they alleviate severe treatment-related side effects [84]. Therefore, targeting gut microbiota-derived metabolites represents a promising therapeutic strategy to enhance tumor immunotherapy response rates in patients.

Research indicates that non-digestible bioactive compounds (e.g., polysaccharides) derived from EMPs are metabolized by specific gut bacteria, such as *Bifidobacterium* and *Lactobacillus*, which subsequently ferment them into short-chain fatty acids (SCFAs), including propionate and butyrate [85,86,87]. SCFAs regulate immune cells function to augment antitumor immune responses and maintain intestinal homeostasis through mechanisms involving the activation of G-protein-coupled receptors (GPRs) and inhibition of histone deacetylases (HDAC) [88,89,90,91]. In addition, (poly)phenols-EMPs components, such as pomegranate ellagitannins, are metabolized by gut microbiota into ellagic acid and further converted into urolithins that can reshape the gut microbiota and modulate the tumor microenvironment. Moreover, when combined with anti-PD-1 antibody therapy, they have been shown to inhibit the growth of colon cancer, thereby supporting more effective anticancer treatment [92,93]. Together, these findings reveal how interactions between gut microbiota and EMPs may offer novel strategies for developing natural adjuvants to enhance cancer immunotherapy.

### 3.3. Intestinal Barrier Dysfunction and Intestinal Inflammation

Cancer immunotherapy, particularly ICIs, commonly triggers immune-related adverse events (irAEs). Many of these irAEs are closely associated with gut microbiota dysbiosis and impaired intestinal barrier function. This impairment, characterized by mucosal injury and increased permeability, facilitates bacterial translocation and sys-temic inflammation [94,95,96,97]. A healthy gut microbiome and an intact intestinal barrier are fundamental to immune homeostasis [98,99]. Their disruption can initiate systemic chronic inflammation, thereby promoting the expansion of immunosuppressive cells, such as myeloid-derived suppressor cells (MDSCs) and regulatory T cells (Tregs). Consequently, an immunosuppressive tumor microenvironment (TME) is created, which can result in either primary or acquired resistance to ICIs [100,101,102]. Therefore, targeting the intestinal barrier and inflammation represents a viable strategy for sensitizing tumors to immunotherapy.

The intestinal barrier functions as a vital line of defense, shielding the body from harmful external pathogens and toxins [103]. It is well established that numerous components from EMPs directly or indirectly contribute to the intestinal barrier integrity [104,105,106]. Polysaccharides, known for their favorable safety profile, are effective in reducing inflammation and suppressing the growth of malignant tumors, which represent a promising adjuvant therapy for a variety of disorders associated with intestinal barrier injury [105]. Numerous bioactive polysaccharides, including those derived from Plantago asiatica [107], Lycium barbarum [108] and Spirulina [109] have been shown to modulate the expression of tight junction proteins (including occludin, claudin-1, ZO-1, and ZO-3), which collectively contribute to the amelioration of epithelial barrier dysfunction.

Additionally, studies indicate that approximately 60% of cancer patients treated with ICIs develop severe treatment-limiting toxicities [110,111], these toxicities are closely linked to inflammation-related adverse events triggered by the treatment [112,113,114]. Colitis is a common and severe adverse effect of ICIs therapy. Therefore, developing targeted therapeutic strategies for intestinal inflammation—aimed at effectively managing toxicity while preserving or even enhancing the antitumor efficacy of ICIs—represents a promising approach. As potent natural anti-inflammatory agents, various bioactive compounds from EMPs serve such as astragalosides [115], curcumin [116,117], ginsenoside Rh2 [118], *Alpinia officinarum* Hance polysaccharides [119], and Portulaca oleracea L. (poly)phenols [120], work synergistically through multiple targets and pathways, precisely modulating key signaling pathways associated with intestinal inflammation, including PI3K/AKT, NF-κB, MAPK, and JAK/STAT—thereby effectively alleviating gut barrier disruption and reducing intestinal inflammation. Clinical studies have also shown that certain medicinal food homologous ingredients, such as curcumin, can serve as an adjunctive therapy to significantly improve the clinical remission rate and endoscopic improvement scores in patients with ulcerative colitis [121].

Based on these findings, it has been elucidated that natural bioactive compounds from EMPs can enhance tumor immunotherapy by modulating the gut microbiota composition, regulating microbial metabolites, improving intestinal barrier dysfunction, and alleviating intestinal inflammation, which work synergistically to mitigate adverse effects induced by immunotherapy, increase patient response rates, and improve overall treatment efficacy.

## 4. Construct DDS Based on Components from EMPs

EMPs compounds including polysaccharides, (poly)phenols, flavonoids, and saponins, demonstrate considerable promise in bolstering tumor immunotherapy by orchestrating the intricate dance between gut microbiota and the immune system. Nevertheless, the journey from lab to clinic for these bioactive compounds is fraught with obstacles, primarily stemming from their inherently low bioavailability. This challenge arises due to their poor water solubility, chemical instability, and inefficient intestinal absorption, which significantly hamper their therapeutic potential. Recent studies have confirmed that advanced drug delivery systems (DDS) strategies, such as nanotechnology, targeted delivery systems, and biotransformation, can significantly enhance the anti-tumor efficacy of EMPs bioactive compounds [122,123,124]. These approaches not only reduce side effects and improve patient compliance but also hold great promise for developing these compounds into effective adjuvant interventions for tumor immunotherapy. However, DDS still faces numerous challenges and problems. EMPs components are now being extensively explored as “smart materials” for constructing or functionalizing drug delivery systems (DDS), rather than merely serving as the encapsulated “cargo,” owing to their inherent bioactivity, excellent biocompatibility, and unique physicochemical properties [125,126]. Additionally, their ability to modulate immune cell function enables an integrated strategy of “delivery and therapy in one.

### 4.1. Advantages of Bioactive Compounds from EMPs for DDS Carriers

**Figure 4:**
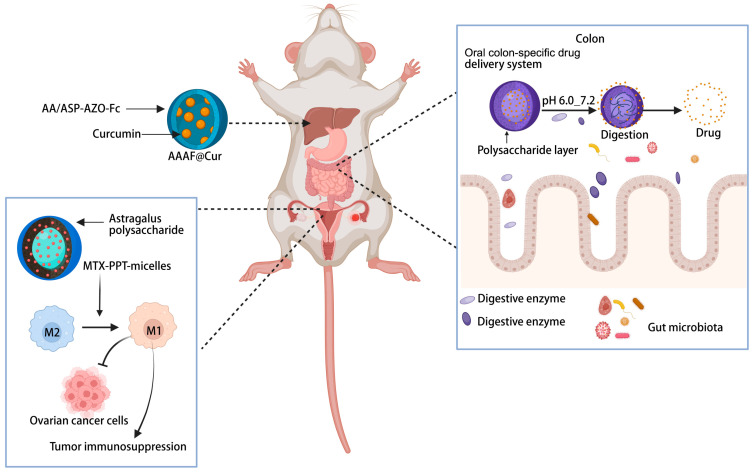
Drug delivery systems constructed from bioactive components of medicinal-edible plants enable precise drug release at specific sites and modulate immune responses against cancer. Created with biorender.com.

An ideal DDS should be capable of precisely delivering drugs to the diseased site. Nevertheless, Dai et al. revealed that due to target heterogeneity, the delivery efficiency of nanoparticles to cancer cells is abysmally low, with only approximately 0.0014% of the ligand-modified nanoparticles successfully binding to them [127]. Natural plant active ingredients such as polysaccharides possess inherent targeting capabilities that can be further optimized through functionalization. By constructing novel DDS using these natural components, it’s possible to significantly improve drug accumulation at the site of disease. For example, Liu et al. developed a novel nanocarrier material, AA/ASP-AZO-Fc, by leveraging the intrinsic liver-targeting property of Angelica sinensis polysaccharide (ASP). The resulting polymeric micelle system effectively delivered curcumin to hepatic tissues, demonstrating a targeted therapeutic strategy for hepatocellular carcinoma in Figure 4 [128]. Furthermore, nanotechnology can be integrated with immunotherapy to enhance tumor-targeted treatment [129]. Liu et al. designed novel methotrexate (MTX)-modified PPT nanoparticles loaded with Astragalus polysaccharide micelles. This system can safely and effectively target ovarian cancer cells and achieve precise drug release. Additionally, it exerts immunomodulatory effects on tumor-associated macrophages to combat tumor cells, thereby enhancing the efficacy of immunotherapy for ovarian cancer [130] (Figure 4).

Indeed, the long-term biosafety of numerous nanocarrier materials is still not fully understood, posing significant concerns for their widespread use in medical applications. Nel and his colleagues emphasized that the inherent characteristics of nano-materials can have adverse impacts, primarily through detrimental interactions with both biological systems and the environment, potentially leading to toxicity [131]. Among these nanomaterials, liposomes have been prominently used as carriers in drug delivery systems. With various formulations available, they have entered clinical applications for targeted cancer therapy [132,133]. However, despite their success, clinical studies have unveiled a crucial dilemma with liposomes: they often exhibit low targeting efficiency and increased systemic toxicity, resulting in unwanted side effects for patients [134,135,136,137]. Natural biopolymers are recognized for their established safety profile, excellent biodegradability, non-toxic degradation products and favorable tolerability, make them promising candidates for replacing synthetic materials in DDS [126,138]. The enhancement of the physicochemical properties of DDS by bioactive compounds such as ginsenosides can increase the bioavailability of active pharmaceutical ingredients, potentiating their antitumor therapeutic effects [126,139]. For instance, ginsenoside Rh2-liposomes can replace cholesterol, significantly prolonging their circulation time in vivo. Their key advantages include active targeting of tumor cells, increased drug accumulation at tumor sites, and effective remodeling of the TME to reverse immunosuppression, potently inhibiting tumor growth [140]. Bioactive food-derived components serving as DDS carriers offer a multifunctional and innovative platform for anticancer drug delivery.

### 4.2. Intestinal-Targeting Release of the Medication

Oral administration remains the most favored method for drug delivery, primarily due to its unparalleled convenience and high patient compliance [141]. However, the highly acidic environment of gastric acid can lead to its inactivation, degradation by gastrointestinal enzymes may reduce its bioavailability, and its premature release in non-target areas could potentially cause systemic side effects [142]. In order to achieve efficient and precise delivery of these active ingredients to specific intestinal sites and control their release behavior, researchers have turned their attention to colon-targeted drug delivery systems. These systems are particularly crucial for the treatment of IBD and colon cancer. They also play a vital role in enhancing the bioavailability of drugs that are prone to degradation, thereby facilitating their systemic absorption [143,144]. Delivery systems utilizing edible bioactive compounds which offer excellent biocompatibility, biodegradability, low toxicity, and ease of functional modification have emerged as ideal carriers for colon-targeted drug delivery [144]. Edible bioactive ingredients exhibit diverse responsive properties, allowing them to be naturally engineered to respond to specific intestinal conditions such as pH and microbial enzymes, enabling targeted drug release at disease sites. Natural polysaccharides are widely used in oral colon-targeted drug delivery systems owing to their high safety, structural diversity, and unique property of being degraded specifically by enzymes produced by the colonic microbiota. This enzymatic reliance enables precise drug release in the colon [145]. Furthermore, leveraging the pH sensitivity of polysaccharides, nanoscale polysaccharide-based carriers can be used to construct responsive delivery systems. This property enables targeted drug release in the intestinal tract and allows for controlled therapeutic delivery [146] (Figure 4). Moreover, orally administered nano-drugs derived from EMPs bioactive components can reach the systemic circulation through colonic absorption. Their antitumor efficacy is then achieved through two primary mechanisms: the direct elimination of tumor cells and the modulation of the gut microbiota [147]. Based on EMPs compounds, natural, safe, and robust delivery systems are expected to lead the field of oral drug delivery into a new era characterized by sustainability, precision, and personalization, thereby offering innovative solutions for nutritional intervention and disease treatment.

## 5. Conclusions and Future Perspectives

In recent years, cancer immunotherapy, particularly ICBs, has become a hotspot in cancer treatment. However, during immunotherapy, due to tumor heterogeneity and inter-patient variability, irAEs might arise, including issues such as drug resistance and poor prognosis. Gut microbiota and their metabolites can modulate tumor immune responses. In turn, cancer can reshape the gut microbial composition, leading to the modulation of TME and the promotion of immune suppression [148]. This review has detailed that EMPs, which are abundant in polysaccharides, (poly)phenols, and saponins (exemplified by traditional sources like ginseng, astragalus, angelica, and turmeric), exhibit favorable characteristics: high safety, stability, low toxicity, and multi-targeting capability. Notably, EMPs can enhance anti-tumor immune responses and synergize with cancer immunotherapy via bidirectional interactions with the gut microbiota, constituting a highly promising interdisciplinary research field. Beyond their direct use as anti-cancer agents, the bioactive compounds of EMPs can also participate in the assembly of drug delivery systems, which enables targeted transport of therapeutic agents to specific sites, facilitating precise cancer treatment. Additionally, DDS assembled from EMPs compounds offer a promising alternative to synthetic materials, mitigating the long-term toxicity concerns associated with their use, while also boosting the bioavailability and anti-tumor potency of the therapeutic agents.

Nevertheless, substantial challenges persist in effectively translating these findings into clinical practice. The inherent variability in the origin, processing, and extraction of EMPs leads to significant inconsistencies in the composition and concentration of their bioactive compounds, resulting in poor reproducibility and unstable research outcomes. This complexity is compounded by the fact that, unlike single-compound drugs, their extracts are multi-component mixtures, making it difficult to determine whether their overall interaction with cancer immunotherapy is synergistic or antagonistic. The translation of bioactive components from EMPs into therapeutic applications faces significant challenges, primarily due to their low solubility, chemical instability, and inefficient absorption. To address these issues, complex delivery systems are necessary. However, individual patient factors, including diet, genetics, and gut microbiome composition, introduce high variability, potentially complicating the outcomes of microbiota-targeted interventions using EMPs bioactive components. Despite the promise of many nano-delivery systems assembled from EMPs bioactive components, their long-term safety, targeting accuracy, and toxicological profile require thorough evaluation. Taken together, these advancements underscore the substantial potential of leveraging the interplay between bioactive components from EMPs and the gut microbiota to enhance the efficacy of cancer immunotherapy.

The bioactive components derived from EMPs offer a promising avenue for enhancing cancer immunotherapy. When combined with traditional treatments like chemotherapy, radiotherapy, and targeted therapy, these components have the potential to mitigate adverse side effects and boost therapeutic outcomes. While preliminary preclinical and small-scale clinical studies have shown encouraging results, further research is imperative. Conducting rigorous clinical trials is crucial to thoroughly assess the safety and effectiveness of these EMPs bioactive components in cancer immunotherapy. Additionally, elucidating the mechanisms of action and comparing their effectiveness with existing treatments will be vital for developing safer, more effective, and personalized cancer therapies. In summary, while the potential of EMPs bioactive constituents in cancer immunotherapy is significant, future research must focus on comprehensive clinical trials, mechanistic elucidation, and comparative effectiveness studies.

## Data Availability

No new data were created or analyzed in this study.
